# Integrated transcriptomics and metabolomics analysis of the hippocampus reveals altered neuroinflammation, downregulated metabolism and synapse in sepsis-associated encephalopathy

**DOI:** 10.3389/fphar.2022.1004745

**Published:** 2022-09-06

**Authors:** Kejia Xu, Hui Li, Bing Zhang, Meini Le, Qiong Huang, Rao Fu, Giorgia Croppi, Gang Qian, Junjie Zhang, Guangming Zhang, Yinzhong Lu

**Affiliations:** ^1^ Department of Anesthesiology and Hongqiao International Institute of Medicine, Tongren Hospital, Shanghai Jiao Tong University School of Medicine, Shanghai, China; ^2^ Department of Anesthesiology, Taihe Hospital, Hubei University of Medicine, Shiyan, China; ^3^ Department of Neurology, Tongren Hospital, Shanghai Jiao Tong University School of Medicine, Shanghai, China; ^4^ Connect Biopharma Ltd, Taicang, China

**Keywords:** sepsis-associated encephalopathy, neuroinflammation, RNA sequencing, metabolomics, integrative analysis, multi-omics analysis

## Abstract

Sepsis-associated encephalopathy (SAE) is an intricated complication of sepsis that brings abnormal emotional and memory dysfunction and increases patients’ mortality. Patients’ alterations and abnormal function seen in SAE occur in the hippocampus, the primary brain region responsible for memory and emotional control, but the underlying pathophysiological mechanisms remain unclear. In the current study, we employed an integrative analysis combining the RNA-seq-based transcriptomics and liquid chromatography/mass spectrometry (LC-MS)-based metabolomics to comprehensively obtain the enriched genes and metabolites and their core network pathways in the endotoxin (LPS)-injected SAE mice model. As a result, SAE mice exhibited behavioral changes, and their hippocampus showed upregulated inflammatory cytokines and morphological alterations. The omics analysis identified 81 differentially expressed metabolites (variable importance in projection [VIP] > 1 and *p* < 0.05) and 1747 differentially expressed genes (Foldchange >2 and *p* < 0.05) were detected in SAE-grouped hippocampus. Moreover, 31 compounds and 100 potential target genes were employed for the Kyoto Encyclopedia of Genes and Genomes (KEGG) Markup Language (KGML) network analysis to explore the core signaling pathways for the progression of SAE. The integrative pathway analysis showed that various dysregulated metabolism pathways, including lipids metabolism, amino acids, glucose and nucleotides, inflammation-related pathways, and deregulated synapses, were tightly associated with hippocampus dysfunction at early SAE. These findings provide a landscape for understanding the pathophysiological mechanisms of the hippocampus in the progression of SAE and pave the way to identify therapeutic targets in future studies.

## Introduction

Sepsis and septic shock are prevalent and severe symptoms in an intensive care unit (ICU). They account for 25%–80% of mortality worldwide (Dellinger et al., 2013; Fleischmann-Struzek et al., 2020; Evans et al., 2021) and usually present with multiorgan dysfunction syndromes, including the heart, kidney, lung, intestine, spleen, and liver. However, the disturbance in peripheral liquid homeostasis causes further damage to the brain, defined as sepsis-associated encephalopathy (SAE), which contributes to the leading cause of death (Young et al., 1990; Eidelman et al., 1996; Schuler et al., 2018). SAE is a severe complication that occurs in 7%–71% of sepsis cases and vastly increases the mortality due to the severity of sepsis, patient status, and diagnosis criteria (Iacobone et al., 2009; Gofton and Young, 2012). The hallmark of SAE is a brain injury, possibly impacting the long-term cognitive (memory loss, attention deficit, or language issues) (Iwashyna et al., 2010; Gofton and Young, 2012; Pandharipande et al., 2013) and psychological functions (depression, anxiety, or post-traumatic stress disorder) (Eidelman et al., 1996; Gofton and Young, 2012; Heming et al., 2017; Neves et al., 2018; Schuler et al., 2018). Although various studies have focused on the diagnosis, clinical progression, and pathological or pathophysiological mechanisms, there are still large gaps in profoundly understanding the mechanisms and identifying the early brain injury and timely intervention for sepsis-suffering patients (Flierl et al., 2010; Gofton and Young, 2012).

Sepsis initiates a series of systemic inflammatory responses leading to deregulated peripheral inflammation, neuroinflammation, ischemic damage, oxidative stress, and metabolic disorders, disrupting the integrity of the brain–blood barrier (BBB) and, thus, causing the progression of SAE (Gofton and Young, 2012). Furthermore, the histological studies on animal models have shown enlarged ventricles, reduced hippocampus volume, and increased apoptosis of hippocampal neurons in lipopolysaccharide (LPS)-exposure rodents (Wang et al., 2013; Chen et al., 2021). Therefore, the hippocampus is an overly sensitive brain region in LPS-triggered animals. Of note, years of studies on experimental sepsis animal models, including LPS injections and cecal-ligation puncture (CLP), have established the dysregulated phenotype of the hippocampus (Hippensteel et al., 2019; Chao et al., 2020; Talukdar et al., 2020) and have been employed to evaluate further the cognitive decline or impairment in the cortex and hippocampus region (Neves et al., 2018; Mei et al., 2021; Shen et al., 2021).

Multiple omics approaches, such as expression array, transcriptomic, and metabolomic, have been employed to elucidate the biological events from different brain regions or spheres to uncover the etiology and molecular mechanisms of cognitive or neurofunction impairment. However, single approaches have gained fewer insights into the complicated mechanisms (Schilder et al., 2022). In contrast, multi-omics analysis is a powerful approach to elucidate the pathophysiological mechanism of diseases, such as neurodegeneration diseases, depression, and other mental diseases (Zhang et al., 2018; Clark et al., 2021; Schilder et al., 2022). Therefore, integrated multi-omics data analysis is promising to identify potential biological relationships and deepen the understanding of SAE.

In the SAE-related study, single omics, including metabolomics and transcriptomics, have been widely used to decipher the molecular mechanisms in the hippocampus (Hara et al., 2015; Salvesen et al., 2017; Geng et al., 2020). Nevertheless, the literature would benefit from more comprehensive study based on multi-omics analysis. Herein, we adopted an RNA-seq and metabolomics-based integrated analysis to explore the possible pathophysiological mechanisms of the hippocampus when mice developed SAE-like symptoms at early stages by an intraperitoneal (i.p.) injection with LPS. The integrated analysis demonstrated that inflammation, neuroinflammation, and synapse and metabolic pathways were mainly altered in LPS-challenge hippocampus tissues.

## Materials and methods

### Materials

All chemicals and solvents were analytical or HPLC-grade. Acetonitrile, methanol, and formic acid were purchased from CNW Technologies GmbH (Düsseldorf, Germany). L-2-chlorophenylalanine was obtained from Shanghai Hengchuang Biotechnology Co., Ltd. (Shanghai, China), and LPS (*Escherichia coli* (O111:B4), #L2630, purity >99%, with high-purity quality Standards M300) was commercially available from Sigma-Aldrich (Saint Louis, MO, United States).

### Animals and LPS injections

C57BL/6J (8–10-week-old male) from SPF facility were purchased from Model Animal Research Center of Nanjing University (China, Nanjing) and maintained in an animal facility with free access to food and *ab libitum* water. Approximately 2 weeks later, a peritoneal injection with LPS saline solution (5 or 10 mg/kg body weight) or saline was randomly administered to mice. All procedures were performed according to regulations for animal experimentation and approved by the ethics committee of Tongren Hospital, Shanghai Jiao Tong University School of Medicine. After 8 h or 24 h post-injection, mice were killed, with the numbers of animals for histology analysis, omics, or biochemical analysis of the hippocampus being described below.

### Open-field test

The open-field test (OFT) widely examined rodent locomotor activity and exploratory behaviors. All open-field testing took place inside an arena (50 cm [l] × 50 cm [w] × 40 cm [h]). The arena was divided into the center and peripheral regions by the VisuTrack system (Shanghai XinRuan Information Technology Co., Ltd., Shanghai, China). Mice that were injected with LPS (10 mg/kg body weight) and controls (injected with saline) for 24 h were removed from their home-cage by the tail and placed directly into the open field center. Tracking/recording was initiated upon the first locomotion grid beam break and lasted 5 min. The trajectory of each mouse was analyzed with the VisuTrack system. Total distance, speed, and freezing time were recorded to evaluate the movement ability of mice, and center entries in the central region were measured to detect mice’s anxiety. The data were interpreted as mean ± SEM (standard error of the mean) (*n* = 9 per group) with Student’s t-test.

### Tissue preparation, nissl staining, and hematoxylin-eosin staining

Twenty-four hours post-injection, mice injected with saline or LPS were anesthetized with 10% chloral hydrate, and whole blood was collected by eyeball sampling before being killed by cervical dislocation. Next, the whole brain was dissected, rinsed with precooled saline, and fixed in 4% paraformaldehyde for paraffin-embedded sections or further dissected for the hippocampi. Then, the dissected hippocampi were snap-frozen by liquid nitrogen and stored at −80°C for subsequent RNA (*n* = 3 per group) and metabolites analysis (*n* = 6 per group). Finally, entire brain coronal sections (5 µm) were used for the Nissl staining or hematoxylin-eosin staining (*n* = 3 mice per group). These stainings were performed as described previously (Xie et al., 2010), scanned by the NDP slide scanner (Hamamatsu NanoZoomer S60), and viewed by its viewing software (NDP.View2). The mean optical density of Nissl staining was measured using Image-Pro Plus 6.0 software (Media Cybernetics, CA, United States) and interpreted as the mean ± SEM.

### RNA extraction and sequencing library construction

Total RNA of each frozen hippocampi was extracted by Trizol reagent (Life-technology, United States) with RNeasy Mini Kit (Qiagen#74106). RNA purity and quantification were evaluated using the NanoDrop 2000 spectrophotometer (Thermo Scientific, United States). RNA integrity was analyzed by Agilent 2100 bioanalyzer (RIN >6.0 and 28S/18S ≥ 0.7); thus, it could qualify for subsequent library construction using NEBNext Ultra RNA Library Prep kit (#E7530; New England BioLabs, Inc., Ipswich, MA, United States) according to the manufacturer’s instructions.

### RNA sequencing and differentially expressed genes analysis

The libraries were sequenced on an Illumina NovaSeq 5,000 platform, and 150-bp paired-end reads were generated. In total, 45.18–49.38 Mb raw reads for each sample were generated. Raw data (raw reads) of fastq format were firstly processed using Trimmomatic (Bolger et al., 2014), and the lower quality reads were removed to obtain the clean reads. Then about 44.21–48.31 Mb clean reads for each sample were retained for subsequent analyses.

The clean reads were mapped to the mouse genome (GRCm38.p6) using HISAT2 (Kim et al., 2015). We calculated the FPKM (Roberts et al., 2011) of each gene by using Cufflinks (Trapnell et al., 2010) and obtained the read counts by HTSeq-count (Simon et al., 2015). Furthermore, we performed differential expression analysis using the DESeq (2012) R package (Anders and Huber, 2013). A P-value of <0.05 and log2 |Foldchange| of >1.0 were set as the threshold for significantly differentially expressed genes (DEGs). Hierarchical cluster analysis of DEGs was performed to demonstrate the expression pattern of genes between the SAE (S) and control (Ctrl) group samples. Gene Ontology (GO) enrichment and Kyoto Encyclopedia of Genes and Genomes (KEGG) (Kanehisa et al., 2008) pathway enrichment analysis of DEGs were performed using R based on the hypergeometric distribution. Bioinformatic analysis was performed using OECloud tools (https://cloud.oebiotech.cn).

Additionally, gene set enrichment analysis (GSEA, http://software.broadinstitute.org/gsea/index.jsp) was used to further screen the signaling pathways associated with SAE (Subramanian et al., 2005). GSEA is a computational method for determining whether a priori-defined set of genes shows statistically significant, concordant differences between two biological states. GSEA does not focus on only significantly/highly changed genes but examines all the genes belonging to a specific biological process.

### Reverse transcription and quantitative polymerase chain reaction

As previously described (Lu et al., 2017; Lu et al., 2021), RNA isolation and RT-qPCR were performed. Briefly, RNA was extracted by Trizol reagent, and 1 μg total RNA was used for cDNA synthesis and reversed transcribed by ReverTra Ace kit (TOYOBO). Then, the cDNA was used to quantify by the lightcycle480 system (Roche) with 2 × Power SYBRgreen mix (Applied Biosystems, Carlsbad, CA, United States). Finally, quantification of gene expression was calculated by normalization to *GAPDH* or *Β-ACTIN* using the 2^−ΔCt^ method. Primer sequences for qPCR are available on request.

### Sample preparation for metabolomics

For sample preparations, 20 μL of internal standard (2-chloro-l-phenylalanine, 0.3 mg/ml in methanol) and 400 μL of methanol: water (4:1 = vol: vol) were added to each hippocampal sample (30 mg/group sample), and ground with a grinder machine (60 Hz, 2 min). Mixtures of the sample were extracted by ultrasonication for 10 min in the ice water bath and kept at −20°C for 30 min. The extraction was centrifuged at 13,000 rpm and 4°C for 10 min. Next, 300 μL of supernatant was transformed into a liquid chromatography/mass spectrometry (LC-MS) injection vial to evaporate and dissolved again in 200 μL methanol: water (4:1 = vol: vol) by sonification (vortex 30 s and sonification 3 min). The samples were kept at −20°C for 2 h and then centrifuged at 13,000 rpm and 4°C for 10 min. The supernatants (150 μL) from each tube were collected using crystal syringes, filtered through 0.22-μm filters, and transferred to LC vials. The vials were stored at −80°C until LC-MS analysis. Of note, all reagents for extraction were precooled at −20°C.

### LC-MS analysis

The liquid chromatography was performed using a Dionex U3000 UHPLC equipped with ACQUITY UPLC HSS T3 column (100 mm × 2.1 mm, 1.8 µm, Waters). The mobile phase consisted of 0.1% formic acid in water (A) and acetonitrile (B). The analysis was performed with elution gradient as follows: 0.0–2.0 min, 5% B; 2.0–4.0 min, 5%–25% B; 4.0–8.0 min, 25%–50% B; 8.0–10.0 min, 50%–80% B; 10.0–14.0 min, 80%–100% B; 14.0–15.0 min, 100% B; 15.0–15.1 min, 100%–5% B, 15.1–16.0 min, 5% B. The column temperature was 45°C. The auto-sampler temperature was 4°C, and the injection volume was 2 ml.

The QE mass spectrometer (ThermoFisher) was used to acquire MS/MS spectra in information-dependent acquisition (IDA) mode in the control of the acquisition software (Xcalibur 4.0.27, Thermofisher). The acquisition software continuously evaluates the full scan MS spectrum in this mode. The ESI source conditions were set as follows: sheath gas flow rate of 35 Arb, Aux gas flow rate of 8 Arb, capillary temperature of 320°C, full MS resolution of 70,000, MS/MS resolution of 17,500, collision energy of 10/20/40 eV in NCE mode, and spray Voltage of 3.8 kV (positive) or −3.0 kV (negative).

### Data preprocessing and analysis

The acquired LC-MS raw data were analyzed by Progenesis QI v2.3 software (Nonlinear Dynamics, Newcastle, United Kingdom) with the following parameters: the precursor tolerance of 5 ppm/10 ppm, fragment tolerance of 10 ppm/20 ppm, and retention time (RT) tolerance of 0.02 min. The product ion threshold was 5%. Internal standard detection parameters were deselected for peak RT alignment, isotopic peaks were excluded for analysis, and the noise elimination level was set at 10.00. The minimum intensity was set to 15% of the base peak intensity. The Excel file was obtained with three-dimension datasets including m/z, peak RT, and peak intensities, and RT-m/z pairs were used as the identifier for each ion. The resulting matrix was further reduced by removing any peaks with a missing value (ion intensity = 0) in >50% of samples. The internal standard was used for data quality control (reproducibility).

Metabolites were identified by progenesis QI software based on public databases, such as HMDB (http://www.hmdb.ca/), Lipidmaps (v2.3) (http://www.lipidmaps.org/), and self-built database (Luming Biotech). The resulting positive and negative ion scans were combined into a dataset for further analysis with R ropls package. Principal component analysis (PCA) and orthogonal partial least-squares-discriminant analysis OPLS-DA were performed to visualize the metabolic alterations among experimental groups. The Hotelling’s T2 region, shown as an ellipse in score plots of the models, defined the 95% confidence interval of the modeled variation. Variable importance in the projection (VIP) ranked the overall contribution of each variable to the OPLS-DA model, and those variables with VIP >1 were considered relevant for group discrimination. In this study, the default 7-round cross-validation was applied, with 1/seventh of the samples being excluded from the mathematical model in each round to guard against overfitting. The criteria for differentially expressed metabolites (DEMs) were VIP of >1.0 obtained from the OPLS-DA model and *p* of <0.05 from a two-tailed Student’s t-test on the normalized peak areas.

### Integrative analysis of transcriptome and metabolome

Building the core genes-metabolites regulation network is better to describe the primary clustered genes and metabolites responsible for the SAE. Therefore, as many studies have shown, we filtered the low expression-abundance DEGs before correlations network analysis and KEGG Markup Language (KGML) network analysis. Pearson correlation coefficients were calculated for transcriptome and metabolome data integration (Luo and Brouwer, 2013). Briefly, based on the DEGs expression data and DEMs concentration data, the Pearson correlation coefficients were calculated by R, and a cluster analysis heatmap of the correlations between the top 100 EMs and DEGs was drawn. Furthermore, DEGs and DEMs were mapped to the KEGG database. KGML, another format storing pathway information in the KEGG database, contained the relationship between all the essential elements in the KEGG pathway and orthologous genes in the KEGG GENES database. Gene, metabolites interaction network, and inter-pathway relationship network were obtained from the KGML file and visualized and interpreted using Cytoscape (version 3.5.1) with MetScape plug-in (version 3.1.3) (Gao et al., 2010).

### Statistical analysis

Statistical analysis was performed on raw data for each group by unpaired Student’s t-test, using GraphPad Prism 8. Data were shown as mean ± SEM. P-values referred to the probability of the null hypothesis meaning that the means do not differ. A P of <0.05 was considered significant, and a p of ≥0.05 was not significant.

## Results

### Proinflammatory cytokines and morphological alterations in the hippocampus with neurobehavior changes in the mice undergo acute sepsis phase

**FIGURE 1:**
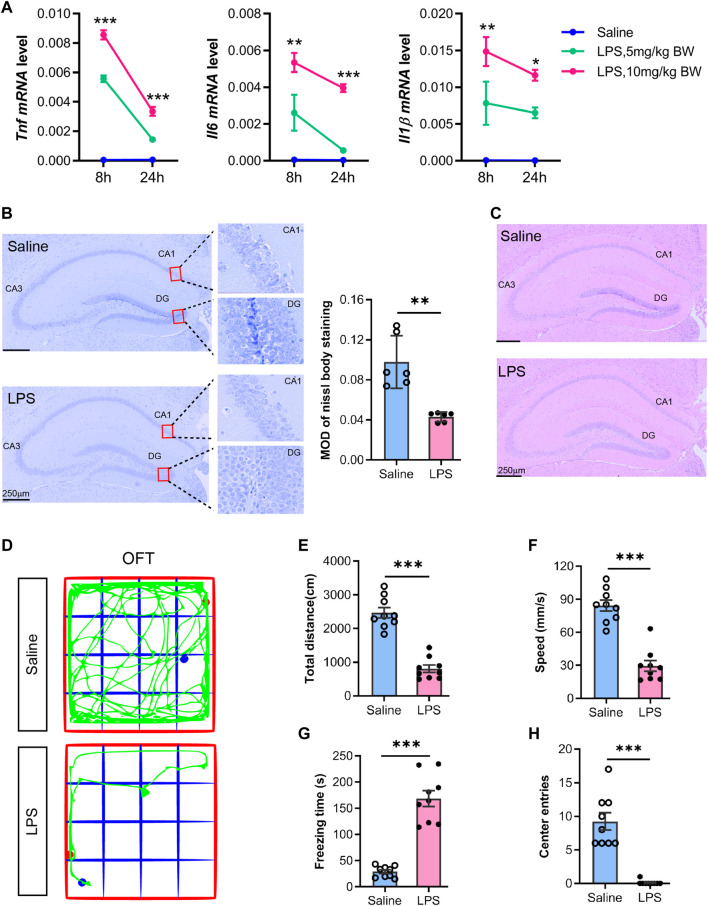
Proinflammatory cytokines and morphological alterations in the hippocampus with neurobehavior changes in mice undergoing acute sepsis phase. **(A)** RT-qPCR analysis of proinflammatory cytokines (*TNF*, *IL6,* and *IL1Β*) alteration in the hippocampus from saline- or LPS-injected (5 or 10 mg/kg body weight) mice. The mRNA expression was normalized to *GAPDH*. Data are presented as mean ± SEM (*n* = 3). Two-way ANOVA with Tukey’s multiple comparisons tests, *p < 0.05, **p < 0.01, ***p < 0.001 (compared with LPS 5 mg/kg body weight at indicated time points). **(B)** Representative Nissl staining of the hippocampus from saline- or LPS-injected mice. The mean optical intensity was quantified and tested with an unpaired *t*-test. Data are presented as mean ± SEM (two sections per mouse, *n* = 6), **p < 0.01. Bar 250 µm. **(C)** Hematoxylin-eosin staining of the hippocampus from saline- or LPS-injected mice (*n* = 3). **(D)** Representative trajectory of the total distance **(E)**, speed **(F)**, freezing time **(G)**, and center entries **(H)** in the LPS mice and saline groups. Comparisons were performed with an unpaired *t*-test. Data are presented as mean ± SEM (*n* = 9), **p < 0.01, ***p < 0.001.

As many studies have shown (Lewis et al., 2016; Savi et al., 2021), we initially constructed the SAE model referring to the sepsis model made by peritoneal injection of a moderate dose of LPS in C57BL6J mice. Then, we evaluated the alterations of cytokines, histology, and behaviors prior to the transcriptomics and metabolomics analysis. The time-chase experiment monitored the dynamic changes of proinflammatory cytokines in the hippocampal tissues by RT-qPCR. The results showed that *Tnf*, *IL1β,* and *Il6* mRNA decreased at 24 h LPS post-injection compared to 8 h (Figure 1A). Furthermore, the cytokine genes in the hippocampus of the LPS-injected group (5 or 10 mg/kg body weight) were more highly expressed than in the saline group. Of note, the high dose of LPS (10 mg/kg body weight) triggered a higher expression of proinflammatory genes than the low dosage. Thus, we determined to use the high dosage for subsequent studies. Furthermore, the histology examination showed a decreased staining in the hippocampal regions, especially the dentate gyrus (DG) region, as showed by Nissl staining (Figure 1B), which indicated hippocampal neurons injury. Hematoxylin and eosin staining showed normal hippocampus morphology in the saline group, whereas minor cell staining showed shrunk and enlarged cells in the adjacent distance among hippocampus regions, especially in the DG region in the LPS group (Figure 1C).

We examined the mice at 24 h post-injection with LPS (10 mg/kg body weight) or saline by open-field test. The results showed behavioral changes, i.e., a decrease in total distance travel, speed, and center entries, but an increase in freezing time (Figure 1D−H) in LPS-injected mice, which was consistent with previous studies with the LPS-sepsis mice model and indicated a potential strike on their cognitive performance (Savi et al., 2021). Most mice demonstrated LPS-manifested back piloerection, noticeably slowed but still ambulant, eyes not fully open, and some were covered with secretions. Furthermore, most mice were stationary with occasional investigative movements and decreased overall activity. The mice also exhibited no response to the auditory stimulus, while they demonstrated strong responses and moved a few steps to escape when touched. Therefore, the animal model was suitable for SAE studies, and we determined to use this model to detect the omics alterations in the hippocampus.

### Non-targeting metabolomic analysis of the hippocampus in acute sepsis phase

**FIGURE 2:**
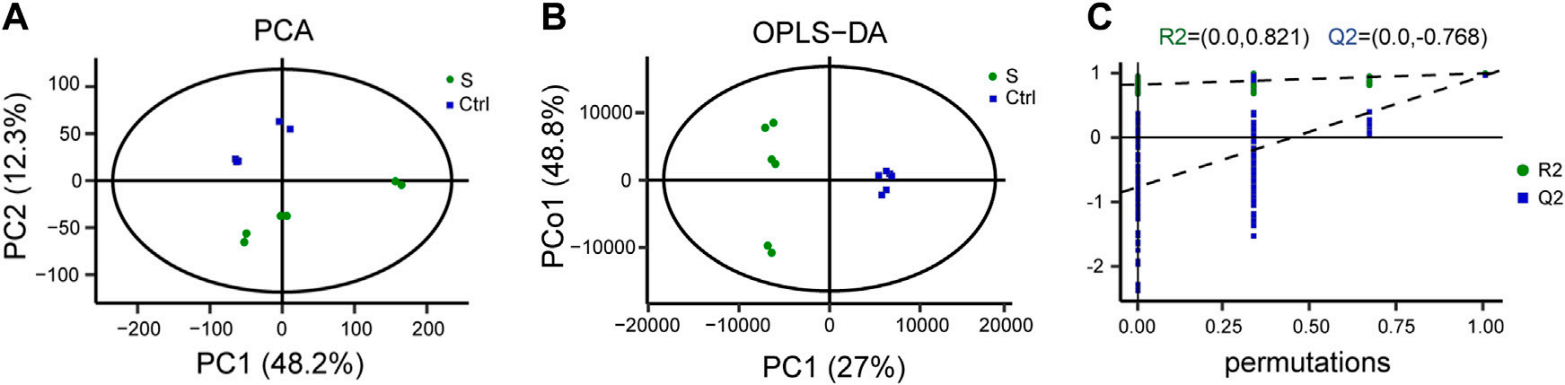
LC-MS-based metabolomic analysis of the hippocampus in the SAE (S) and control groups (Ctrl). **(A)** Principal component analysis (PCA) scores plot (*n* = 6). **(B)** Ortholog partial least squares-discriminate analysis (OPLS-DA) scores. **(C)** The permutation plot.

We performed LC-MS-based metabolomics profiling of the hippocampus in the SAE model and control groups (*n* = 6 mice per group) to determine the alterations of metabolites. A total of 5,109 metabolites (2133 and 2976 identified in positive and negative ion mode, respectively) were detected by LC-MS and subsequently used for multivariate analysis. The PCA and OPLS-DA score plot showed that the SAE group was discriminated from the saline group (Figures 2A,B). Furthermore, the permutation tests showed no overfitting of data (Figure 2C), indicating that the OPLS-DA model was valid for the analyses.

**FIGURE 3:**
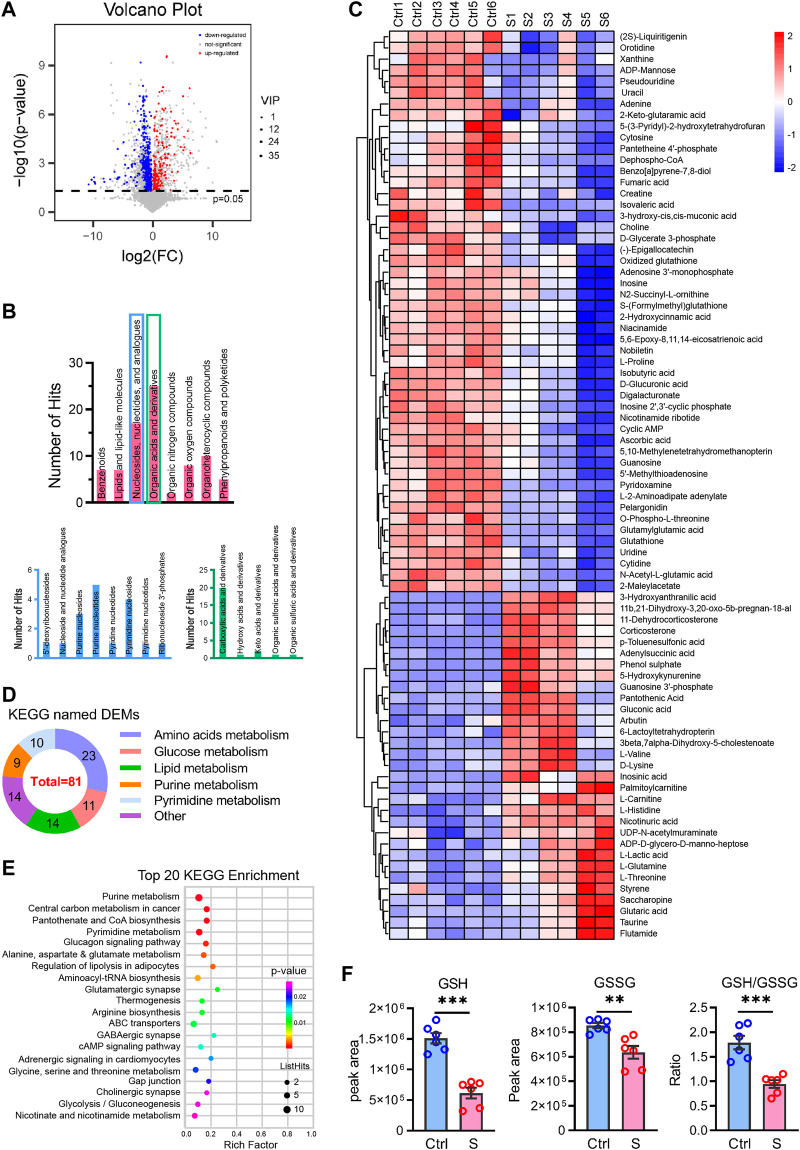
Differentially expressed metabolites profiling the hippocampus in the SAE (S) and control groups (Ctrl). **(A)** Volcano plot showing differentially expressed metabolites (DEMs) profiling of hippocampus between the S and the Ctrl group, the red and blue bubble showed the up- and downregulated metabolites, respectively. **(B)** The classes of differential expressed metabolites in the SAE-grouped hippocampus. **(C)** The heatmap showed the relative contents of DEMs. S1–S6, the sepsis group; Ctrl1– 6, the control group. **(D)** The classes of metabolism of DEMs in the KEGG (*n* = 3). **(E)** The top 20 KEGG enrichment items of DEMs. **(F)** The peak area of GSH and GSSG, and the ratio of GSH/GSSG, Statistic comparisons were performed by unpaired t-test. Data are presented as mean ± SEM (*n* = 6), *p < 0.05, **p < 0.01.

We then performed the OPLS-DA test and identified 149 upregulated and 199 downregulated DEMs between the SAE group (S1−S6) and the control group (Ctrl1−6) under the criteria of VIP >1 and *p* < 0.05 (Figure 3A). Of these DEMs, 81 were categorized in the KEGG database and classified into eight superclasses, such as lipids and lipid-like molecules (*n* = 7), nucleosides, nucleotides, and analogs (n = 17), organic acids and derivatives (*n* = 25), and others (Figure 3B). They are listed in Table 1 and contained 31 upregulated and 50 downregulated DEMs. Their expression is over-presented in the heatmap (Figure 3C), which shows their involvement in amino acid metabolism (*n* = 23), glucose metabolism (*n* = 11), lipid metabolism (*n* = 14), purine metabolism (*n* = 11) and pyrimidine metabolism (*n* = 8), and other uncategorized metabolisms (Figure 3D; Table 1).

**TABLE 1:** Differentially expressed metabolites in the hippocampus between sepsis-associated encephalopathy and control group.

No.	Metabolites	Ion mode	KEGG	Formula	VIP	P-value	FC^a^	Pathway mainly involved
1	Glutamylglutamic acid	pos	C01425	C10H16N2O7	13.77	7.9E-08	0.52	Amino acid metabolism
2	Creatine	pos	C00300	C4H9N3O2	12.92	6.9E-03	0.94	Amino acid metabolism
3	L-Glutamine	pos	C00064	C5H10N2O3	12.23	1.2E-03	1.49	Amino acid metabolism
4	Glutathione	pos	C00051	C10H17N3O6S	11.90	2.6E-05	0.41	Amino acid metabolism
5	Oxidized glutathione	pos	C00127	C20H32N6O12S2	5.57	2.7E-03	0.74	Amino acid metabolism
6	Taurine	pos	C00245	C2H7NO3S	5.21	4.0E-02	1.47	Amino acid metabolism
7	L-Valine	pos	C00183	C5H11NO2	5.11	2.3E-02	1.36	Amino acid metabolism
8	L-Proline	pos	C00148	C5H9NO2	3.68	7.1E-03	0.81	Amino acid metabolism
9	L-Histidine	pos	C00135	C6H9N3O2	3.16	8.8E-06	1.25	Amino acid metabolism
10	L-Threonine	pos	C00188	C4H9NO3	2.78	4.1E-04	1.51	Amino acid metabolism
11	D-Lysine	pos	C00739	C6H14N2O2	2.68	8.0E-03	1.14	Amino acid metabolism
12	Pantetheine 4′-phosphate	neg	C01134	C11H23N2O7PS	2.54	3.7E-03	0.39	Amino acid metabolism
13	N2-Succinyl-L-ornithine	pos	C03415	C9H16N2O5	2.22	1.2E-02	0.69	Amino acid metabolism
14	O-Phospho-L-threonine	neg	C12147	C4H10NO6P	2.13	2.1E-04	0.80	Amino acid metabolism
15	3-Hydroxyanthranilic acid	pos	C00632	C7H7NO3	2.11	1.1E-05	5.27	Amino acid metabolism
16	N-Acetyl-L-glutamic acid	pos	C00624	C7H11NO5	1.96	6.4E-06	0.74	Amino acid metabolism
17	S-(Formylmethyl)glutathione	neg	C14871	C12H19N3O7S	1.44	2.5E-03	0.61	Amino acid metabolism
18	2-Keto-glutaramic acid	neg	C00940	C5H7NO4	1.36	1.4E-02	0.87	Amino acid metabolism
19	Glutaric acid	pos	C00489	C5H8O4	1.27	2.4E-02	1.20	Amino acid metabolism
20	5-Hydroxykynurenine	pos	C05651	C10H12N2O4	1.27	1.0E-05	60.03	Amino acid metabolism
21	Nicotinuric acid	pos	C05380	C8H8N2O3	1.19	1.4E-06	1.37	Amino acid metabolism
22	Saccharopine	pos	C00449	C11H20N2O6	1.14	5.8E-03	3.90	Amino acid metabolism
23	5′-Methylthioadenosine	neg	C00170	C11H15N5O3S	1.09	8.1E-04	0.61	Amino acid metabolism
24	D-Glucuronic acid	neg	C00191	C6H10O7	11.13	6.7E-04	0.56	Glucose metabolism
25	Ascorbic acid	neg	C01041	C6H8O6	9.76	1.7E-03	0.48	Glucose metabolism
26	Pantothenic Acid	pos	C00864	C9H17NO5	6.05	1.4E-02	1.36	Glucose metabolism
27	Gluconic acid	neg	C00257	C6H12O7	3.03	1.4E-03	2.48	Glucose metabolism
28	ADP-D-glycero-D-manno-heptose	pos	C06397	C17H27N5O16P2	3.01	5.1E-03	1.81	Glucose metabolism
29	Digalacturonate	neg	C02273	C12H18O13	2.90	9.8E-04	0.48	Glucose metabolism
30	L-Lactic acid	neg	C00186	C3H6O3	2.83	9.5E-03	1.26	Glucose metabolism
31	Adenylsuccinic acid	pos	C03794	C14H18N5O11P	2.74	8.0E-04	2.21	Glucose metabolism
32	ADP-Mannose	pos	C06192	C16H25N5O15P2	1.15	4.8E-03	0.22	Glucose metabolism
33	Fumaric acid	neg	C00122	C4H4O4	1.01	8.2E-04	0.50	Glucose metabolism
34	D-Glycerate 3-phosphate	neg	C00197	C3H7O7P	1.00	1.2E-04	0.66	Glucose metabolism
35	Choline	pos	C00114	C5H13NO	6.83	6.5E-04	0.88	Lipid metabolism
36	(-)-Epigallocatechin	neg	C12136	C15H14O7	6.57	2.4E-03	0.72	Lipid metabolism
37	L-Carnitine	pos	C00318	C7H15NO3	5.62	3.4E-04	1.19	Lipid metabolism
38	Palmitoylcarnitine	pos	C00547	C23H45NO4	5.02	4.2E-03	3.11	Lipid metabolism
39	Nobiletin	pos	C10112	C21H22O8	4.67	1.6E-03	0.56	Lipid metabolism
40	Corticosterone	pos	C02140	C21H30O4	3.32	7.9E-05	7.59	Lipid metabolism
41	5,6-Epoxy-8,11,14-eicosatrienoic acid	neg	C14768	C20H32O3	2.62	8.3E-03	0.69	Lipid metabolism
42	Isovaleric acid	pos	C08262	C5H10O2	1.61	3.6E-05	0.16	Lipid metabolism
43	Isobutyric acid	neg	C02632	C4H8O2	1.50	6.3E-04	0.55	Lipid metabolism
44	11-Dehydrocorticosterone	pos	C05490	C21H28O4	1.27	8.0E-05	3.36	Lipid metabolism
45	3β,7α-Dihydroxy-5-cholestenoate	pos	C17335	C27H44O4	1.16	1.4E-03	3.29	Lipid metabolism
46	11β,21-Dihydroxy-3,20-oxo-5β-pregnan-18-al	pos	C05473	C21H32O5	1.12	3.6E-06	20.12	Lipid metabolism
47	3-hydroxy-cis,cis-muconic acid	pos	C03676	C6H6O5	1.11	1.6E-03	0.45	Lipid metabolism
48	Pelargonidin	neg	C05904	C15H11O5+	1.08	6.4E-06	0.44	Lipid metabolism
49	Inosine	neg	C00294	C10H12N4O5	18.85	3.6E-02	0.77	Purine metabolism
50	Guanosine	neg	C00387	C10H13N5O5	4.37	9.2E-04	0.68	Purine metabolism
51	Xanthine	pos	C00385	C5H4N4O2	3.96	3.4E-02	0.78	Purine metabolism
52	Guanosine 3′-phosphate	pos	C06193	C10H14N5O8P	2.76	1.6E-02	1.31	Purine metabolism
53	Adenine	pos	C00147	C5H5N5	2.74	1.5E-03	0.42	Purine metabolism
54	Dephospho-CoA	neg	C00882	C21H35N7O13P2S	1.34	4.3E-03	0.19	Purine metabolism
55	L-2-Aminoadipate adenylate	neg	C05560	C16H23N6O10P	1.30	1.5E-04	0.36	Purine metabolism
56	Cyclic AMP	neg	C00575	C10H12N5O6P	1.19	1.1E-03	0.45	Purine metabolism
57	Adenosine 3′-monophosphate	pos	C01367	C10H14N5O7P	1.11	4.4E-02	0.83	Purine metabolism
58	Uridine	neg	C00299	C9H12N2O6	8.67	2.2E-04	0.60	Pyrimidine metabolism
59	Cytidine	pos	C00475	C9H13N3O5	5.56	8.1E-05	0.64	Pyrimidine metabolism
60	Inosinic acid	neg	C00130	C10H13N4O8P	5.35	4.3E-04	4.83	Pyrimidine metabolism
61	Pseudouridine	pos	C02067	C9H12N2O6	3.85	5.9E-04	0.68	Pyrimidine metabolism
62	Uracil	pos	C00106	C4H4N2O2	3.01	3.2E-03	0.81	Pyrimidine metabolism
63	UDP-N-acetylmuraminate	pos	C01050	C20H31N3O19P2	1.90	4.7E-03	1.31	Pyrimidine metabolism
64	Orotidine	neg	C01103	C10H12N2O8	1.61	1.4E-03	0.67	Pyrimidine metabolism
65	Cytosine	pos	C00380	C4H5N3O	1.47	2.8E-02	0.80	Pyrimidine metabolism
66	Nicotinamide ribotide	neg	C00455	C11H15N2O8P	1.27	1.8E-03	0.42	Pyrimidine metabolism
67	Pyridoxamine	pos	C00534	C8H12N2O2	1.13	9.3E-06	0.22	Pyrimidine metabolism
68	Niacinamide	pos	C00153	C6H6N2O	10.71	3.9E-03	0.76	Other
69	2-Hydroxycinnamic acid	pos	C01772	C9H8O3	6.14	1.6E-02	0.78	Other
70	Styrene	pos	C19506	C8H8	2.17	4.3E-02	1.22	Other
71	Inosine 2′,3′-cyclic phosphate	neg	C05768	C10H11N4O7P	2.14	8.9E-05	0.35	Other
72	Phenol sulphate	neg	C02180	C6H6O4S	1.91	2.6E-05	13.13	Other
73	p-Toluenesulfonic acid	neg	C06677	C7H8O4S	1.85	8.3E-06	18.90	Other
74	(2S)-Liquiritigenin	neg	C09762	C15H12O4	1.67	2.4E-03	0.45	Other
75	Benzo[a]pyrene-7,8-diol	neg	C14852	C20H14O2	1.51	4.6E-03	0.46	Other
76	2-Maleylacetate	pos	C02222	C6H6O5	1.45	3.1E-05	0.55	Other
77	Arbutin	pos	C06186	C12H16O7	1.43	4.3E-03	1.59	Other
78	5,10-Methylenetetrahydromethanopterin	neg	C04377	C31H45N6O16P	1.31	3.2E-04	0.36	Other
79	6-Lactoyltetrahydropterin	pos	C04244	C9H13N5O3	1.23	4.5E-03	2.80	Other
80	Flutamide	pos	C07653	C11H11F3N2O3	1.16	3.9E-02	1.28	Other
81	5-(3-Pyridyl)-2-hydroxytetrahydrofuran	pos	C19578	C9H11NO2	1.06	2.9E-02	0.39	Other

VIP, varible importance in the projection; FC, foldchange.

a FC was calculated as the ratio of sepsis grouped hippocampus to the control grouped hippocampus.

The DEMs matched in the KEGG database were significantly enriched in glycolysis/gluconeogenesis, regulation of lipolysis, amino acids metabolism, nucleotide metabolism (purine metabolism, pyrimidine metabolism), synapse function (glutamatergic, GABAergic, and cholinergic synapse, and ABC transporters), and signal transduction (glucagon signaling pathway, cAMP signaling pathway, adrenergic signaling) (Figure 3E). Additionally, the GSH/GSSG redox was altered in the SAE-grouped hippocampus (Figure 3F).

### Transcriptome and DEGs analysis of the hippocampus in the early stage of SAE mice

We next performed RNA-seq-based profiles analysis using the same batch of hippocampus tissues as in LC-MS metabolomics analysis to uncover the molecular basis of the hippocampus from SAE mice. A total of 41703102–4411552 new reads were sequenced, mapped into the mouse genome, and deposited in the NCBI sequence read archive (SRA, BioProject accession number PRJNA827615).

**FIGURE 4:**
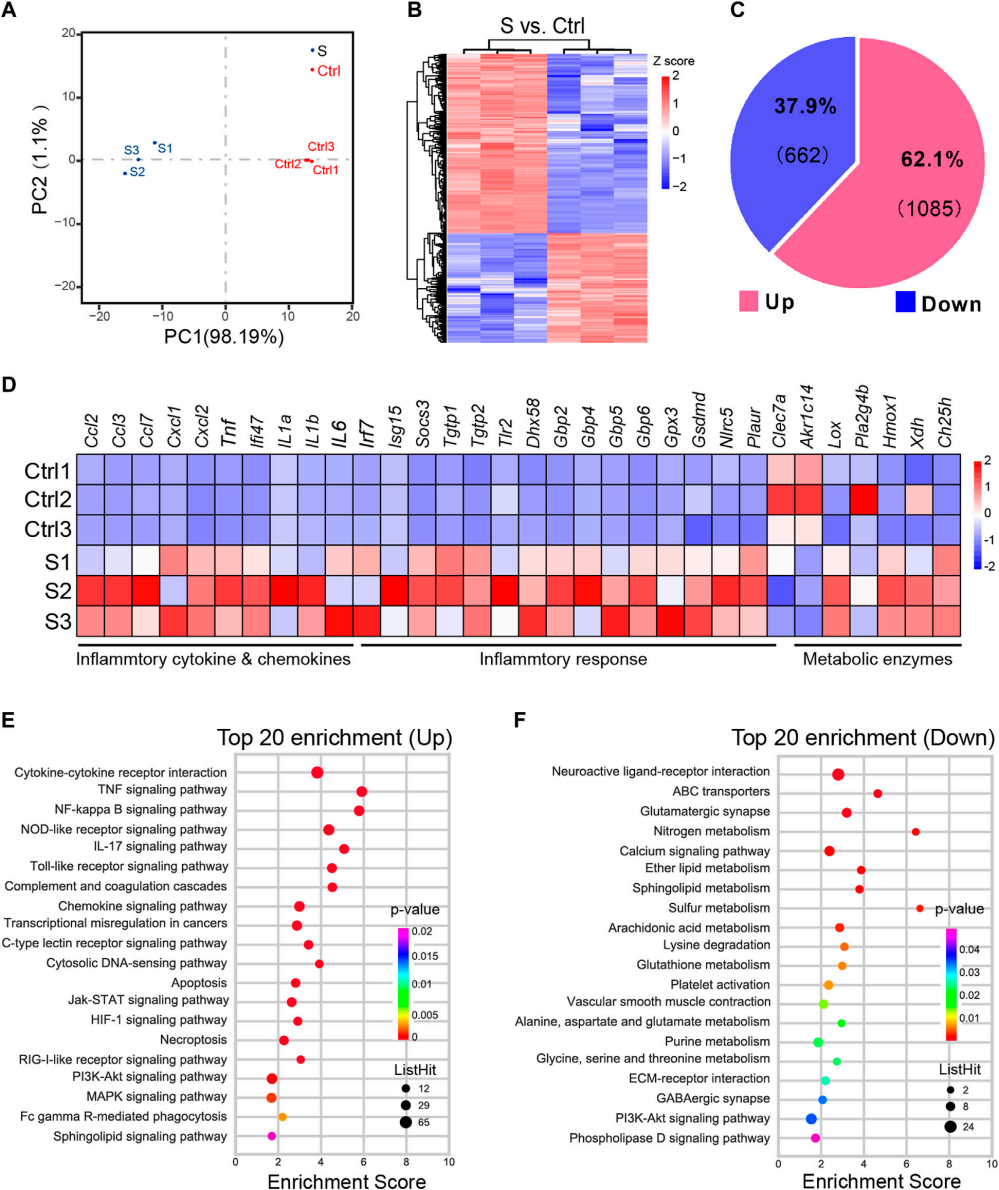
Differentially expressed genes profiling of the hippocampus in the SAE (S) and control groups (Ctrl). **(A)** The PCA analysis of hippocampus from SAE- and Ctrl-grouped mice (*n* = 3). **(B)** The heatmap of differentially expressed genes (DEGs) with a cutoff log2FC of >1 and p of <0.05 (*n* = 3). **(C)** The pie chart illustrating the composition of up- and downregulated DEGs. **(D)** Heatmap of RT-qPCR analysis showing upregulation of inflammatory cytokines and chemokines, inflammatory response, and metabolic enzymes gene expression in the hippocampal tissues, i.p., injected with LPS (*n* = 3). **(E,F)** The top 20 KEGG enrichment of upregulated **(E)** or downregulated DEGs **(F)**.

The PCA analysis of the hippocampus transcriptome signature showed clear segregation between SAE (S1−S3, *n* = 3) and control (Ctrl 1–3, *n* = 3) (Figure 4A). Next, we made a DESeq analysis to determine the significant DEGs with a cutoff |Log2FC| of > 1 and p of < 0.05 (Figure 4B) and identified 662 upregulated and 1085 downregulated DEGs in the SAE of interest (Figure 4C; Supplementary Table S1). Moreover, a pool of 32 DEGs related to inflammatory cytokines and chemokines, inflammatory response, and metabolic enzyme genes was confirmed by RT-qPCR analysis (Figure 4D). These data showed consistency in the expression pattern between control and SAE hippocampus tissues.

**FIGURE 5:**
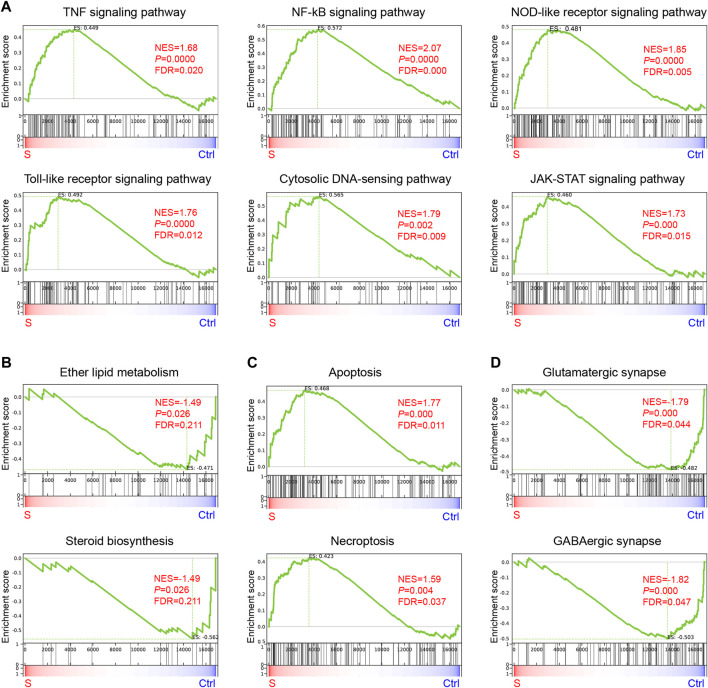
Gene enrichment analysis of genes enriched in the hippocampus during the progression of SAE. **(A–D)** KEGG Terms related to inflammation **(A)**, lipid metabolism (B), apoptosis and necroptosis **(C)**, and synapse **(D)**.

KEGG analysis was performed to obtain the top 20 significant pathway terms with the upregulated or downregulated DEGs. For the upregulated DEGs, the KEGG term included most of the inflammatory response, cytokine signaling pathways, necroptosis, and apoptosis in the hippocampus (Figure 4E; Supplementary Figure S1). In addition to the downregulated DEGs, the metabolism (nitrogen, sphingolipid, ether lipid, glutathione, alanine, aspartate and glutamate, glycine, serine and threonine, purine, sulfur, arachidonic acid, and lysine degradation), synapse function (glutamatergic synapse and GABAergic synapse), and signaling pathways (e.g., neuroactive ligand-receptor interaction, calcium signaling pathway, ECM-receptor interaction, PI3K-Akt signaling pathway, and phospholipase D signaling pathway) were enriched (Figure 4F and Supplementary Figure S2), indicating that the metabolism was suppressed in SAE-grouped hippocampus. Furthermore, GSEA analysis (Subramanian et al., 2005) of the biological event (Figure 5 and Supplementary Table S2) further corroborated these top-ranked inflammatory response-related pathways, lipid metabolism, cell death, and synapse.

### Integrative analysis of transcriptome and metabolome

**FIGURE 6:**
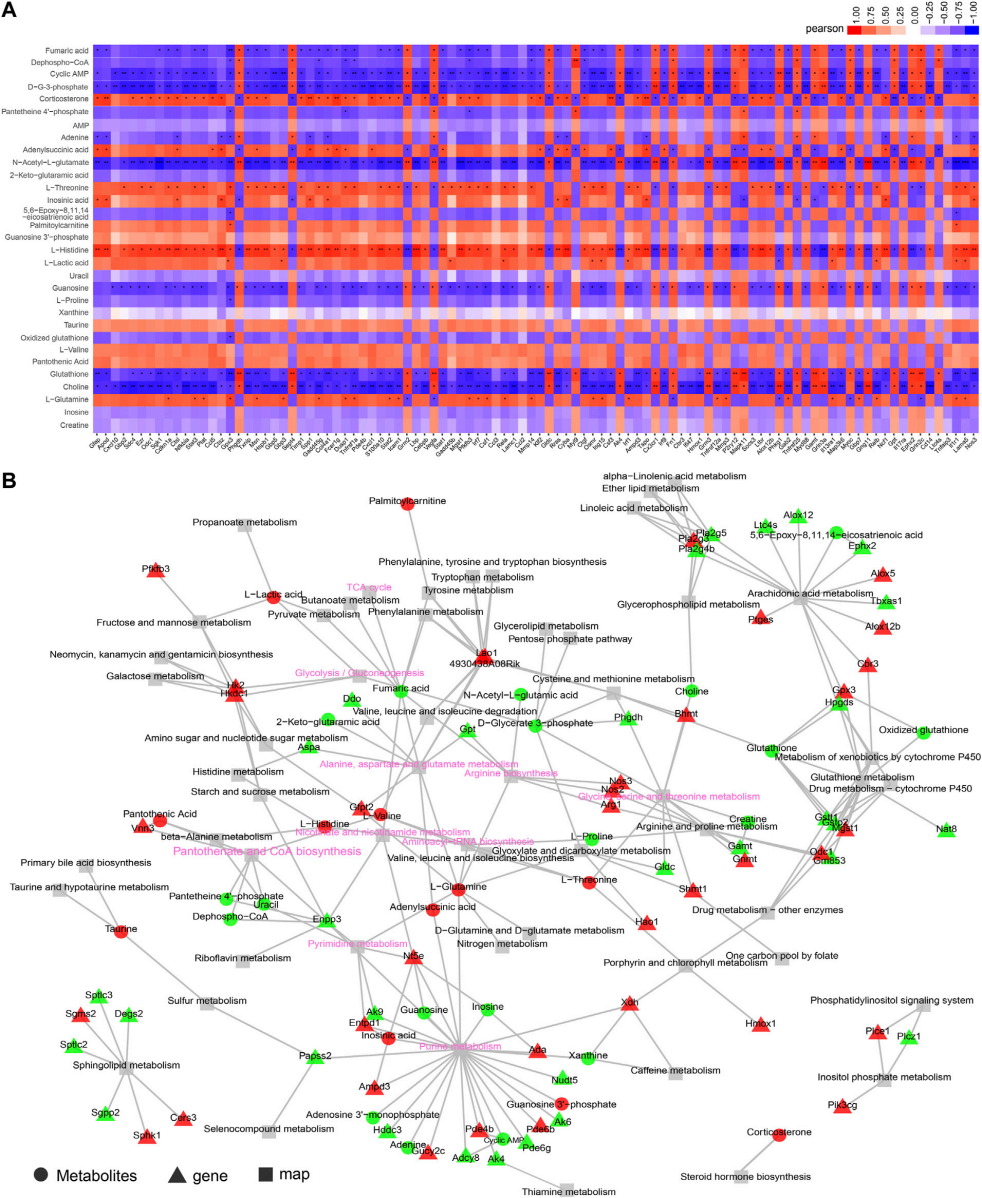
Integrative analysis of transcriptome and metabolome. **(A)** Correlation heatmap of links between top 100 expressed genes and metabolites based on the Pearson correlation algorithm. The color bar denotes the value of correlation coefficiency, *p < 0.05, **p < 0.01, ***p < 0.001. **(B)** KGML network building based on the DEGs and DEMs to obtain the critical nodes and metabolism pathways. The triangle with red or green denotes the up- or downregulated DEGs, respectively. The circle with red or green denotes the up- or downregulated DEMs, respectively, and the square denotes the pathway. The key metabolic or signaling pathways were shown with pink fonts.

A core genes-metabolites regulation network must decipher the significant clustered genes and metabolites responsible for the SAE progression. According to the KEGG enrichment analysis with the RNA-seq and metabolomics data, we observed that 392 DEGs and 31 DEMs in a cluster of signaling pathways were highly represented in the SAE group. The top 100 expression DEGs and top 100 DEMs (only 31 DEMs) were used for Pearson correlation to distinguish the regulations among the DEGs and DEMs. The correlation heatmap shows the calculated correlation coefficient results (Figure 6A and Supplementary Table S3). Additionally, the KGML network was built based on the 100 DEGs and 31DEMs datasets to gain the global network information during SAE progression. The results showed that the critical nodes and metabolism pathways were highly over-presented (Figure 6B).

## Discussion

The development of acute sepsis in patients can cause septic shock and further develop as SAE (Iacobone et al., 2009; Gofton and Young, 2012). In this study, we examined the enriched expressed genes and metabolites of the hippocampus to decipher the possible mechanisms of SAE in the early phase with an LPS-induced experimental sepsis model. The results showed that acute sepsis mice developed inflammation (dramatic upregulation of proinflammatory cytokines), hippocampal neuron injury, and behavior changes, which indicated that a reproducible SAE-like-symptom occurred in hippocampal tissues as in previous findings (Wang et al., 2013; Chen et al., 2021).

Metabolomics has been vigorously used to explore the enriched metabolites and related metabolic pathways in the development of SAE (Hara et al., 2015; Salvesen et al., 2017; Geng et al., 2020), which leads to the discovery of biomarkers or sensitive indicators to detect their pathophysiological activity. However, the metabolomic profiles only illuminate a landscape of metabolic alterations during the progression of SAE. Additionally, the gene expression profiles most uniquely reflect the protein levels that directly regulate the metabolic process. Therefore, a multi-omics analysis could offer a more comprehensive understanding of the molecular regulatory network. According to the literature, no reports on the hippocampus in SAE were documented with multi-omics analysis. Interestingly, this study integrated two different omics data to identify functional associations between them and pinpointed pathways that were perturbed among the lipid metabolic, amino acid, and carbohydrate pathways of the hippocampus of the SAE model.

### Lipids metabolism alterations in the hippocampus of SAE

The lipids altered in sepsis have been documented in many studies (Amunugama et al., 2021; Preau et al., 2021). In the present study, the lipids metabolism-related pathways were highly enriched in the hippocampus of SAE by integrative analysis of metabolomics data and RNA-seq data. Furthermore, the lipid metabolism-related pathways were enriched by using the DEMs, which were further corroborated by top-ranked pathways, including ether lipid metabolism, sphingolipid metabolism, arachidonic acid metabolism, phospholipase D signaling pathway, and sphingolipid signaling pathway as enriched by DEGs (Figures 4, 5).

Steroids (corticosterone [CORT], 11-dehydrocorticosterone [11-DHC], 3β, 7α-dihydroxy-5-cholestenoate, and 11β, 21-dihydroxy-3, 20-oxo-5β-pregnan-18-al) were accumulated in the hippocampus of SAE. Moreover, lipids and steroids metabolism-related genes were detected to be upregulated (*ALOX5*, *CYP1B1*, *CYP27B1*, *CYP3A13*, *PLA2G4E*, *SOAT2*, *UGT1A6A*, and *UGT1A7C*) or downregulated (*DHCR24*, *DHCR7*, *CYP11A1*, *PLA2G4B*, *SOAT1*, and *SRD5A1*) in the hippocampus tissues of SAE (Supplementary Table S1, Figure 3C). Those results indicated that steroid synthesis-related pathways were significantly affected and consistent with previous findings that the CORT was increased in murine sepsis survivors and exhibited a behavioral neuroendocrine syndrome (Spencer-Segal et al., 2020). Furthermore, CORT was reported to induce neuroinflammation and bring depression-like behaviors or other neuron dysfunction syndromes in the hippocampus (Komoltsev et al., 2021; Bras et al., 2022), which indicated that the increase of CORT is a critical factor in the progression of SAE. Additionally, other enriched steroids were precursors of CORT, but their functions are still unknown.

Apart from the upregulated steroids, 5,6-epoxy-8,11,14-eicosatrienoic acids (5, 6-EET) are tightly related to arachidonic acid metabolism and isovaleric acid, which is associated with cognitive impairment and systemic inflammation (Zheng et al., 2022), and were decreased in the SAE-grouped hippocampus (Table 1 and Figure 3C). This result indicated that their anti-inflammatory effects were blocked, which might cause further cognitive impairment in the hippocampus during the progression of SAE (Dalli et al., 2017; Wang et al., 2021). The mentioned steroids induced inflammation, further corroborated with the enriched inflammation-related pathways, including the tumor necrosis factor (TNF) signaling pathway, nuclear factor kappa B (NF-κB) signaling pathway, nucleotide oligomerization domain (NOD)-like receptor signaling pathway, toll-like receptor signaling pathway, and chemokine signaling pathway as top-ranked pathways by RNA-seq analysis. RT-qPCR confirmed that several genes were a part of the inflammatory mediators driving inflammation. *PLA2G3*, *CH25H*, *NLRC5*, and *PLAUR* (Murase et al., 2017; Wang et al., 2019; Gonias, 2021; Izumi et al., 2021; Rasmussen et al., 2021) were upregulated, while *CLEC7A* (Deerhake and Shinohara, 2021) was downregulated in the hippocampus of SAE (Figure 4D; Supplementary Table S1), and they can also affect the neurological function (Rasmussen et al., 2021). The PLA2 family members of inflammation mediators participate in glycerophospholipid metabolism (Sun et al., 2005) and affect choline production. The sphingolipid signaling pathway was another enriched lipid metabolism-related pathway in our studies. Consistent with previous findings, it regulates the BBB in the LPS-injected mice model for SAE (Vutukuri et al., 2018). Therefore, the altered lipids mediate inflammation or neuroinflammation in the hippocampus during the progression of SAE.

### Amino acid metabolism alterations in the hippocampus of SAE

Amino acid metabolism was disturbed in the hippocampus of SAE in gas chromatography (GC)-MS-based metabolomics analyses (Geng et al., 2020). In light of this, more altered amino acids and derivatives, including the increase in L-glutamine, taurine, L-valine, L-histidine, L-threonine, D-lysine, glutaric acid, 5-hydroxykynurenine, nicotinuric acid, and saccharopine and the reduction in creatine, glutathione, oxidized glutathione, L-proline, pantetheine 4′-phosphate, 2-keto-glutaramic acid, and 5′-methylthioadenosine, in the hippocampus of SAE was detected in our study, which belong to several metabolisms of amino acids, and this difference among them was likely to explain by diverse methods of metabolomics analysis.

The increase in L-glutamine, taurine, L-valine, L-histidine, L-threonine, and D-lysine has been found in the plasma of sepsis patients and correlated to the severity of sepsis (Chiarla et al., 2011), indicating that the hippocampus has similar metabolic adaptations with peripheral tissues. The saccharopine degradation pathway is the primary route of lysine degradation, and accumulated saccharopine impairs normal neuron development (Guo et al., 2022). The increase in glutamine leads to decreased 2-keto-glutaramic acid, indicating that glutamine metabolism is deregulated in SAE conditions. Glutaric acid accumulation is linked to neurodegeneration (Rodrigues et al., 2013; Rodrigues et al., 2019). 5-hydroxykynurenine, as the precursor of serotonin, is involved in the serotonin pathway, which produces the neurotransmitter serotonin and affects emotional status (Palacios-Filardo and Mellor, 2019). Creatine and choline were decreased in the hippocampus of patients with mild cognitive impairment (Tumati et al., 2013), indicating their associations with cognitive impairment in SAE. It is reported that taurine has versatile effects in modulating neuroinflammatory activity, cellular redox homeostasis, Ca^2+^ homeostasis, and neurogenesis (Jakaria et al., 2019). Therefore, the increased taurine in SAE indicates a feedback metabolism regulation to the neurological dysfunction. Because 5′-methylthioadenosine is a metabolite that has anti-inflammatory effects in preventing endotoxin-induced lethality and inflammatory response (Hevia et al., 2004), its decreased level would deteriorate the inflammation of the hippocampus in SAE.

Glutathione metabolism and the GSH: GSSG redox homeostasis is critical to maintaining neuron physiology (Aoyama, 2021). Interestingly, both glutathione (GSH) and oxidized glutathione (GSSG) were decreased in the hippocampus of SAE mice. However, we observed that the ratio of GSH/GSSG was 1:1.85 (Figure 3F), which indicated that GSH: GSSG redox homeostasis was disturbed in the hippocampus due to sepsis. Collectively, the amino acid metabolism-related pathway, as mentioned above, would disrupt the redox homeostasis, drive inflammatory response, and dampen the anti-inflammatory effects in the hippocampus during the early stage of SAE progression.

### Glucose metabolism and nucleotide metabolism alterations in the hippocampus of SAE

D-glycerate 3-phosphate (G3P) decreased and subsequently increased L-lactic acid production, indicating that glycolysis was reinforced for a metabolic adaptation to the inflammatory response in the SAE-grouped hippocampus. However, several metabolites (fumaric acid and ascorbic acid) holding anti-inflammatory and/or neuroprotective effects were decreased, and the oxidative stress metabolite gluconic acid was increased in the SAE group (Linker et al., 2011; Ament et al., 2021).

Regarding the nucleotides, the level of purine nucleobases (guanosine and adenine) and pyridine nucleobases (uridine, cytidine, and cytosine) decreased in the SAE hippocampus, indicating that their pyrimidine pathway was impaired and in shortage of energy. Uracil has been shown as the intermediate metabolite to neuroprotection and neuroinflammation (Lecca and Ceruti, 2008). Adenine and pseudouridine could cause inflammation; however, other nucleotides and derivatives (inosine, guanosine, pyridoxamine, and cyclic AMP) have anti-inflammatory effects (Gerbatin et al., 2017; Itokawa et al., 2018; Veremeyko et al., 2018; Lovaszi et al., 2021) that decrease as SAE progresses. This result suggested that the metabolites could not affect the anti-inflammatory response. Adenosine 3′-monophosphate (AMP, for ATP synthesis), cyclic AMP (cAMP, second messenger), and dephospho-CoA (for CoA synthesis) were downregulated in the SAE hippocampus. Uracil, guanosine, pyridoxamine, and cAMP could be pivotal to neuron repair and neuroprotection (Lecca and Ceruti, 2008; Gerbatin et al., 2017; Itokawa et al., 2018; Veremeyko et al., 2018), indicating that their supplementation might be a therapeutic strategy in the treatment of SAE. Additionally, the metabolisms mentioned above corroborated by the enrichment with downregulated DEGs in the hippocampus of SAE (Figure 4F) and suggested that the glycolysis was enhanced. The nucleotides metabolism also decreased, which indicated an insufficient energy supply disturbing the balance between anabolic and catabolic processes to fuel the neurons and minimize the metabolic brain injury, i.e., the metabolisms of purine, amino acids, and lipids turned into the catabolic process.

### Deregulated synapse and behaviors in the hippocampus of SAE

Behavior abnormality and cognitive dysfunction are common characteristics of SAE progression (Eidelman et al., 1996; Gofton and Young, 2012; Heming et al., 2017; Neves et al., 2018; Schuler et al., 2018). Synaptic plasticity relies on the hippocampus synapses normal functions, thus, forming the long-term potentiation (LTP) to improve learning and memory (Magee and Grienberger, 2020). In the present study, we noted several altered metabolites, such as L-glutamine, choline, and cAMP, that were enriched in the synapses-related biological events including glutamatergic synapse, GABAergic synapse, cholinergic synapse, and the calcium signaling pathways (Figure 3), were most affected and deregulated in SAE-grouped hippocampus, which possibly explained the deterioration of locomotion, exploration, and anxiety-like behaviors that likely implicated learning and memory during the progression of SAE. Additionally, other metabolites, such as CORT, steroids (5,6-EET and isovaleric acid), amino acids (glutaric acid, creatine, and choline), and nucleotides, may directly or indirectly affect behavior (Lecca and Ceruti, 2008; Rodrigues et al., 2013; Tumati et al., 2013; Palacios-Filardo and Mellor, 2019; Rodrigues et al., 2019; Spencer-Segal et al., 2020; Komoltsev et al., 2021; Bras et al., 2022), for example, CORT induce a depressive- or seize-like behaviors via neuroinflammation dysfunction (Komoltsev et al., 2021; Bras et al., 2022). Furthermore, several signaling pathways affected neuron plasticity, including the PI3K-Akt signaling, calcium signaling pathway, and cAMP signaling pathways, and were enriched as the top-ranked pathways in the SAE-grouped hippocampus by RNA-seq analysis (Figure 4). These findings indicated that the dysregulated synapse functions were linked to the behavioral alterations in early SAE. However, further studies need to monitor the long periods of SAE behaviors to corroborate the early phase changes.

Although our study depicted that dysregulated neuroinflammation, metabolism, and synapse pathways were tightly associated with the hippocampus at the early SAE, several key predictions, such as the lipid mediator, and amino acids, need further functional validation. Furthermore, it is still a challenge to directly monitor the cognitive behaviors of SAE rodents at the early stages (Savi et al., 2021) because they move infrequently and are manifested by other alterations in peripheral organ systems or other brain regions at this stage. Additionally, the maturated LPS-sepsis model advanced our studies, but the different sepsis models vary in their mortality and clinic associations (Lewis et al., 2016). Besides, the gender difference was closely correlated with prognosis of sepsis, as the estrogen protective role in inflammation (Kondo et al., 2021). Therefore, future work needs to consider other SAE models including in female rodents and monitor their behavior changes, genes, and metabolites in the middle or late stage of SAE.

In summary, RNA-seq-based transcriptomics and LC-MS-based metabolomics were gathered for the first time to obtain a comprehensive landscape of the hippocampus of SAE. Our integrated studies showed that various dysregulated metabolism pathways, including lipids metabolism, amino acid, glucose, and nucleotide metabolism, inflammation-related pathways, and deregulated synapses were possible mechanisms of SAE progression in the hippocampus. This study provided comprehensive omics data analysis to understand the possible pathophysiological mechanism of the hippocampus in the early phase of SAE. Furthermore, it might pioneer the SAE field to identify future therapeutic targets for brain protection.

## Data Availability

The datasets of RNA-seq have been deposited in sequence read archive (SRA) and assigned a BioProject accession number PRJNA827615. Further reasonable requests for raw data should be directed to the corresponding authors.

## Supplementary material

Supplementary Figure S1 Heatmap analysis of inflammation-related pathways and cell death.

Supplementary Figure S2 Heatmap analysis of the metabolism of the lipids, amino acids and nucleotides, and synapse.

Supplementary Table S1 List of differentially expressed genes.

Supplementary Table S2 GSEA analysis of biological events in the hippocampus of SAE.

Supplementary Table S3 Correlation of top100 differential mRNA-differential metabolite.

## References

[B1] Ament Z. Bevers M. B. Wolcott Z. Kimberly W. T. Acharjee A. (2021). Uric acid and gluconic acid as predictors of hyperglycemia and cytotoxic injury after stroke. Transl. Stroke Res. 12 (2), 293–302. 10.1007/s12975-020-00862-5 33067777 PMC7933067

[B2] Amunugama K. Pike D. P. Ford D. A. (2021). The lipid biology of sepsis. J. Lipid Res. 62, 100090. 10.1016/j.jlr.2021.100090 34087197 PMC8243525

[B3] Anders S. Huber W. (2012). Differential expression of RNA-Seq data at the gene level - the DESeq package[J]. Heidelberg,Germany: European Molecular Biology Laboratory (EMBL).

[B4] Aoyama K. (2021). Glutathione in the brain. Int. J. Mol. Sci. 22 (9), 5010. ARTN. 10.3390/ijms22095010 34065042 PMC8125908

[B5] Bolger A. M. Lohse M. Usadel B. (2014). Trimmomatic: a flexible trimmer for Illumina sequence data. Bioinformatics 30 (15), 2114–2120. 10.1093/bioinformatics/btu170 24695404 PMC4103590

[B6] Bras J. P. Guillot de Suduiraut I. Zanoletti O. Monari S. Meijer M. Grosse J. (2022). Stress-induced depressive-like behavior in male rats is associated with microglial activation and inflammation dysregulation in the hippocampus in adulthood. Brain Behav. Immun. 99, 397–408. 10.1016/j.bbi.2021.10.018 34793941

[B7] Chao R. Ren-Qi Y. Hui Z. Yong-Wen F. Yong-Ming Y. (2020). Sepsis-associated encephalopathy: a vicious cycle of immunosuppression. J. Neuroinflammation 17 (1), 14. 10.1186/s12974-020-1701-3 31924221 PMC6953314

[B8] Chen Y. Li L. Zhang J. Cui H. Wang J. Wang C. (2021). Dexmedetomidine alleviates lipopolysaccharide-induced hippocampal neuronal apoptosis via inhibiting the p38 MAPK/c-Myc/CLIC4 signaling pathway in rats. Mol. Neurobiol. 58 (11), 5533–5547. 10.1007/s12035-021-02512-9 34363182

[B9] Chiarla C. Giovannini I. Siegel J. H. (2011). Characterization of alpha-amino-n-butyric acid correlations in sepsis. Transl. Res. 158 (6), 328–333. 10.1016/j.trsl.2011.06.005 22061040

[B10] Clark C. Dayon L. Masoodi M. Bowman G. L. Popp J. (2021). An integrative multi-omics approach reveals new central nervous system pathway alterations in Alzheimer's disease. Alzheimers Res. Ther. 13 (1), 71. 10.1186/s13195-021-00814-7 33794997 PMC8015070

[B11] Dalli J. Colas R. A. Quintana C. Barragan-Bradford D. Hurwitz S. Levy B. D. (2017). Human sepsis eicosanoid and proresolving lipid mediator temporal profiles: Correlations with survival and clinical outcomes. Crit. Care Med. 45 (1), 58–68. 10.1097/CCM.0000000000002014 27632672 PMC5549882

[B12] Deerhake M. E. Shinohara M. L. (2021). Emerging roles of Dectin-1 in noninfectious settings and in the CNS. Trends Immunol. 42 (10), 891–903. 10.1016/j.it.2021.08.005 34489167 PMC8487984

[B13] Dellinger R. P. Levy M. M. Rhodes A. Annane D. Gerlach H. Opal S. M. (2013). Surviving sepsis campaign: International guidelines for management of severe sepsis and septic shock, 2012. Intensive Care Med. 39 (2), 165–228. 10.1007/s00134-012-2769-8 23361625 PMC7095153

[B14] Eidelman L. A. Putterman D. Putterman C. Sprung C. L. (1996). The spectrum of septic encephalopathy. Definitions, etiologies, and mortalities. JAMA 275 (6), 470–473. 10.1001/jama.1996.03530300054040 8627969

[B15] Evans L. Rhodes A. Alhazzani W. Antonelli M. Coopersmith C. M. French C. (2021). Surviving sepsis campaign: International guidelines for management of sepsis and septic shock 2021. Intensive Care Med. 47 (11), 1181–1247. 10.1007/s00134-021-06506-y 34599691 PMC8486643

[B16] Fleischmann-Struzek C. Mellhammar L. Rose N. Cassini A. Rudd K. E. Schlattmann P. (2020). Incidence and mortality of hospital- and ICU-treated sepsis: results from an updated and expanded systematic review and meta-analysis. Intensive Care Med. 46 (8), 1552–1562. 10.1007/s00134-020-06151-x 32572531 PMC7381468

[B17] Flierl M. A. Rittirsch D. Huber-Lang M. S. Stahel P. F. (2010). Pathophysiology of septic encephalopathy--an unsolved puzzle. Crit. Care 14 (3), 165. 10.1186/cc9035 20565858 PMC2911737

[B18] Gao J. Tarcea V. G. Karnovsky A. Mirel B. R. Weymouth T. E. Beecher C. W. (2010). Metscape: a Cytoscape plug-in for visualizing and interpreting metabolomic data in the context of human metabolic networks. Bioinformatics 26 (7), 971–973. 10.1093/bioinformatics/btq048 20139469 PMC2844990

[B19] Geng C. Guo Y. Wang C. Cui C. Han W. Liao D. (2020). Comprehensive evaluation of lipopolysaccharide-induced changes in rats based on metabolomics. J. Inflamm. Res. 13, 477–486. 10.2147/JIR.S266012 32904659 PMC7457572

[B20] Gerbatin R. D. R. Cassol G. Dobrachinski F. Ferreira A. P. O. Quines C. B. Pace I. D. D. (2017). Guanosine protects against traumatic brain injury-induced functional impairments and neuronal loss by modulating excitotoxicity, mitochondrial dysfunction, and inflammation. Mol. Neurobiol. 54 (10), 7585–7596. 10.1007/s12035-016-0238-z 27830534

[B21] Gofton T. E. Young G. B. (2012). Sepsis-associated encephalopathy. Nat. Rev. Neurol. 8 (10), 557–566. 10.1038/nrneurol.2012.183 22986430

[B22] Gonias S. L. (2021). Plasminogen activator receptor assemblies in cell signaling, innate immunity, and inflammation. Am. J. Physiol. Cell Physiol. 321 (4), C721–C734. 10.1152/ajpcell.00269.2021 34406905 PMC8560384

[B23] Guo Y. Wu J. Wang M. Wang X. Jian Y. Yang C. (2022). The metabolite saccharopine impairs neuronal development by inhibiting the neurotrophic function of glucose-6-phosphate isomerase. J. Neurosci. 42 (13), 2631–2646. 10.1523/JNEUROSCI.1459-21.2022 35135854 PMC8973428

[B24] Hara N. Chijiiwa M. Yara M. Ishida Y. Ogiwara Y. Inazu M. (2015). Metabolomic analyses of brain tissue in sepsis induced by cecal ligation reveal specific redox alterations--protective effects of the oxygen radical scavenger edaravone. Shock 44 (6), 578–584. 10.1097/SHK.0000000000000465 26529662

[B25] Heming N. Mazeraud A. Verdonk F. Bozza F. A. Chrétien F. Sharshar T. (2017). Neuroanatomy of sepsis-associated encephalopathy. Crit. Care 21 (1), 65. 10.1186/s13054-017-1643-z 28320461 PMC5360026

[B26] Hevia H. Varela-Rey M. Corrales F. J. Berasain C. Martinez-Chantar M. L. Latasa M. U. (2004). 5'-methylthioadenosine modulates the inflammatory response to endotoxin in mice and in rat hepatocytes. Hepatology 39 (4), 1088–1098. 10.1002/hep.20154 15057913

[B27] Hippensteel J. A. Anderson B. J. Orfila J. E. McMurtry S. A. Dietz R. M. Su G. (2019). Circulating heparan sulfate fragments mediate septic cognitive dysfunction. J. Clin. Invest. 129 (4), 1779–1784. 10.1172/JCI124485 30720464 PMC6436867

[B28] Iacobone E. Bailly-Salin J. Polito A. Friedman D. Stevens R. D. Sharshar T. (2009). Sepsis-associated encephalopathy and its differential diagnosis. Crit. Care Med. 37 (10), S331–S336. 10.1097/CCM.0b013e3181b6ed58 20046118

[B29] Itokawa M. Miyashita M. Arai M. Dan T. Takahashi K. Tokunaga T. (2018). Pyridoxamine: A novel treatment for schizophrenia with enhanced carbonyl stress. Psychiatry Clin. Neurosci. 72 (1), 35–44. 10.1111/pcn.12613 29064136

[B30] Iwashyna T. J. Ely E. W. Smith D. M. Langa K. M. (2010). Long-term cognitive impairment and functional disability among survivors of severe sepsis. JAMA 304 (16), 1787–1794. 10.1001/jama.2010.1553 20978258 PMC3345288

[B31] Izumi Y. Cashikar A. G. Krishnan K. Paul S. M. Covey D. F. Mennerick S. J. (2021). A proinflammatory stimulus disrupts hippocampal plasticity and learning via microglial activation and 25-hydroxycholesterol. J. Neurosci. 41 (49), 10054–10064. 10.1523/JNEUROSCI.1502-21.2021 34725187 PMC8660051

[B32] Jakaria M. Azam S. Haque M. E. Jo S. H. Uddin M. S. Kim I. S. (2019). Taurine and its analogs in neurological disorders: Focus on therapeutic potential and molecular mechanisms. Redox Biol. 24, 101223. 10.1016/j.redox.2019.101223 31141786 PMC6536745

[B33] Kanehisa M. Araki M. Goto S. Hattori M. Hirakawa M. Itoh M. (2008). KEGG for linking genomes to life and the environment. Nucleic Acids Res. 36, D480–D484. 10.1093/nar/gkm882 18077471 PMC2238879

[B34] Kim D. Langmead B. Salzberg S. L. (2015). HISAT: a fast spliced aligner with low memory requirements. Nat. Methods 12 (4), 357–360. 10.1038/nmeth.3317 25751142 PMC4655817

[B35] Komoltsev I. G. Frankevich S. O. Shirobokova N. I. Volkova A. A. Onufriev M. V. Moiseeva J. V. (2021). Neuroinflammation and neuronal loss in the Hippocampus are associated with immediate posttraumatic seizures and corticosterone elevation in rats. Int. J. Mol. Sci. 22 (11), 5883. 10.3390/ijms22115883 34070933 PMC8198836

[B36] Kondo Y. Miyazato A. Okamoto K. Tanaka H. (2021). Impact of sex differences on mortality in patients with sepsis after trauma: A nationwide cohort study. Front. Immunol. 12, 678156. 10.3389/fimmu.2021.678156 34267751 PMC8276106

[B37] Lecca D. Ceruti S. (2008). Uracil nucleotides: from metabolic intermediates to neuroprotection and neuroinflammation. Biochem. Pharmacol. 75 (10), 1869–1881. 10.1016/j.bcp.2007.12.009 18261711

[B38] Lewis A. J. Seymour C. W. Rosengart M. R. (2016). Current murine models of sepsis. Surg. Infect. 17 (4), 385–393. 10.1089/sur.2016.021 PMC4960474 27305321

[B39] Linker R. A. Lee D. H. Ryan S. van Dam A. M. Conrad R. Bista P. (2011). Fumaric acid esters exert neuroprotective effects in neuroinflammation via activation of the Nrf2 antioxidant pathway. Brain 134 (3), 678–692. 10.1093/brain/awq386 21354971

[B40] Lovaszi M. Nemeth Z. H. Gause W. C. Beesley J. Pacher P. Hasko G. (2021). Inosine monophosphate and inosine differentially regulate endotoxemia and bacterial sepsis. FASEB J. 35 (11), e21935. 10.1096/fj.202100862R 34591327 PMC9812230

[B41] Lu Y. Wan J. Yang Z. Lei X. Niu Q. Jiang L. (2017). Regulated intramembrane proteolysis of the AXL receptor kinase generates an intracellular domain that localizes in the nucleus of cancer cells. FASEB J. 31 (4), 1382–1397. 10.1096/fj.201600702R 28034848 PMC5349800

[B42] Lu Y. Zhang W. Zhang B. Heinemann S. H. Hoshi T. Hou S. (2021). Bilirubin oxidation end products (BOXes) induce neuronal oxidative stress involving the Nrf2 pathway. Oxid. Med. Cell. Longev. 2021, 8869908. 10.1155/2021/8869908 34373769 PMC8349295

[B43] Luo W. Brouwer C. (2013). Pathview: an R/bioconductor package for pathway-based data integration and visualization. Bioinformatics 29 (14), 1830–1831. 10.1093/bioinformatics/btt285 23740750 PMC3702256

[B44] Magee J. C. Grienberger C. (2020). Synaptic plasticity forms and functions. Annu. Rev. Neurosci. 43, 95–117. 10.1146/annurev-neuro-090919-022842 32075520

[B45] Mei B. Li J. Zuo Z. (2021). Dexmedetomidine attenuates sepsis-associated inflammation and encephalopathy via central α2A adrenoceptor. Brain Behav. Immun. 91, 296–314. 10.1016/j.bbi.2020.10.008 33039659 PMC7749843

[B46] Murase R. Taketomi Y. Miki Y. Nishito Y. Saito M. Fukami K. (2017). Group III phospholipase A2 promotes colitis and colorectal cancer. Sci. Rep. 7 (1), 12261. 10.1038/s41598-017-12434-z 28947740 PMC5612992

[B47] Neves F. S. Marques P. T. Barros-Aragao F. Nunes J. B. Venancio A. M. Cozachenco D. (2018). Brain-defective insulin signaling is associated to late cognitive impairment in post-septic mice. Mol. Neurobiol. 55 (1), 435–444. 10.1007/s12035-016-0307-3 27966074

[B48] Palacios-Filardo J. Mellor J. R. (2019). Neuromodulation of hippocampal long-term synaptic plasticity. Curr. Opin. Neurobiol. 54, 37–43. 10.1016/j.conb.2018.08.009 30212713 PMC6367596

[B49] Pandharipande P. P. Girard T. D. Jackson J. C. Morandi A. Thompson J. L. Pun B. T. (2013). Long-term cognitive impairment after critical illness. N. Engl. J. Med. 369 (14), 1306–1316. 10.1056/NEJMoa1301372 24088092 PMC3922401

[B50] Preau S. Vodovar D. Jung B. Lancel S. Zafrani L. Flatres A. (2021). Energetic dysfunction in sepsis: a narrative review. Ann. Intensive Care 11 (1), 104. 10.1186/s13613-021-00893-7 34216304 PMC8254847

[B51] Rasmussen L. J. H. Caspi A. Ambler A. Danese A. Elliott M. Eugen-Olsen J. (2021). Association between elevated suPAR, a new biomarker of inflammation, and accelerated aging. J. Gerontol. A Biol. Sci. Med. Sci. 76 (2), 318–327. 10.1093/gerona/glaa178 32766674 PMC7812430

[B52] Roberts A. Trapnell C. Donaghey J. Rinn J. L. Pachter L. (2011). Improving RNA-Seq expression estimates by correcting for fragment bias. Genome Biol. 12 (3), R22. 10.1186/gb-2011-12-3-r22 21410973 PMC3129672

[B53] Rodrigues F. S. de Zorzi V. N. Funghetto M. P. Haupental F. Cardoso A. S. Marchesan S. (2019). Involvement of the cholinergic parameters and glial cells in learning delay induced by glutaric acid: Protection by N-acetylcysteine. Mol. Neurobiol. 56 (7), 4945–4959. 10.1007/s12035-018-1395-z 30421167

[B54] Rodrigues F. S. Souza M. A. Magni D. V. Ferreira A. P. Mota B. C. Cardoso A. M. (2013). N-acetylcysteine prevents spatial memory impairment induced by chronic early postnatal glutaric acid and lipopolysaccharide in rat pups. PLoS One 8 (10), e78332. 10.1371/journal.pone.0078332 24205200 PMC3813430

[B55] Salvesen O. Reiten M. R. Espenes A. Bakkebo M. K. Tranulis M. A. Ersdal C. (2017). LPS-induced systemic inflammation reveals an immunomodulatory role for the prion protein at the blood-brain interface. J. Neuroinflammation 14 (1), 106. 10.1186/s12974-017-0879-5 28532450 PMC5441080

[B56] Savi F. F. de Oliveira A. de Medeiros G. F. Bozza F. A. Michels M. Sharshar T. (2021). What animal models can tell us about long-term cognitive dysfunction following sepsis: A systematic review. Neurosci. Biobehav. Rev. 124, 386–404. 10.1016/j.neubiorev.2020.12.005 33309906

[B57] Schilder B. M. Navarro E. Raj T. (2022). Multi-omic insights into Parkinson's Disease: From genetic associations to functional mechanisms. Neurobiol. Dis. 163, 105580. 10.1016/j.nbd.2021.105580 34871738 PMC10101343

[B58] Schuler A. Wulf D. A. Lu Y. Iwashyna T. J. Escobar G. J. Shah N. H. (2018). The impact of acute organ dysfunction on long-term survival in sepsis. Crit. Care Med. 46 (6), 843–849. 10.1097/CCM.0000000000003023 29432349 PMC5953770

[B59] Shen Y. Zhang Y. Du J. Jiang B. Shan T. Li H. (2021). CXCR5 down-regulation alleviates cognitive dysfunction in a mouse model of sepsis-associated encephalopathy: potential role of microglial autophagy and the p38MAPK/NF-κB/STAT3 signaling pathway. J. Neuroinflammation 18 (1), 246. 10.1186/s12974-021-02300-1 34711216 PMC8554863

[B60] Simon A. Theodor P. P. Wolfgang H. (2015). HTSeq—a Python framework to work with high-throughput sequencing data. Bioinformatics 31 (2), 166–169. 10.1093/bioinformatics/btu638 25260700 PMC4287950

[B61] Spencer-Segal J. L. Singer B. H. Laborc K. Somayaji K. Watson S. J. Standiford T. J. (2020). Sepsis survivor mice exhibit a behavioral endocrine syndrome with ventral hippocampal dysfunction. Psychoneuroendocrinology 117, 104679. 10.1016/j.psyneuen.2020.104679 32353815 PMC7845932

[B62] Subramanian A. Tamayo P. Mootha V. K. Mukherjee S. Ebert B. L. Gillette M. A. (2005). Gene set enrichment analysis: a knowledge-based approach for interpreting genome-wide expression profiles. Proc. Natl. Acad. Sci. U. S. A. 102 (43), 15545–15550. 10.1073/pnas.0506580102 16199517 PMC1239896

[B63] Sun G. Y. Xu J. Jensen M. D. Yu S. Wood W. G. Gonzalez F. A. (2005). Phospholipase A2 in astrocytes: responses to oxidative stress, inflammation, and G protein-coupled receptor agonists. Mol. Neurobiol. 31 (1-3), 27–41. 10.1385/MN:31:1-3:027 15953810

[B64] Talukdar P. M. Abdul F. Maes M. Binu V. S. Venkatasubramanian G. Kutty B. M. (2020). Maternal immune activation causes schizophrenia-like behaviors in the offspring through activation of immune-inflammatory, oxidative and apoptotic pathways, and lowered antioxidant defenses and neuroprotection. Mol. Neurobiol. 57 (10), 4345–4361. 10.1007/s12035-020-02028-8 32720073

[B65] Trapnell C. Williams B. A. Pertea G. Mortazavi A. Kwan G. van Baren M. J. (2010). Transcript assembly and quantification by RNA-Seq reveals unannotated transcripts and isoform switching during cell differentiation. Nat. Biotechnol. 28 (5), 511–515. 10.1038/nbt.1621 20436464 PMC3146043

[B66] Tumati S. Martens S. Aleman A. (2013). Magnetic resonance spectroscopy in mild cognitive impairment: systematic review and meta-analysis. Neurosci. Biobehav. Rev. 37 (10), 2571–2586. 10.1016/j.neubiorev.2013.08.004 23969177

[B67] Veremeyko T. Yung A. W. Y. Dukhinova M. Kuznetsova I. S. Pomytkin I. Lyundup A. (2018). Cyclic AMP pathway suppress autoimmune neuroinflammation by inhibiting functions of encephalitogenic CD4 T cells and enhancing M2 macrophage polarization at the site of inflammation. Front. Immunol. 9, 50. 10.3389/fimmu.2018.00050 29422898 PMC5788911

[B68] Vutukuri R. Brunkhorst R. Kestner R. I. Hansen L. Bouzas N. F. Pfeilschifter J. (2018). Alteration of sphingolipid metabolism as a putative mechanism underlying LPS-induced BBB disruption. J. Neurochem. 144 (2), 172–185. 10.1111/jnc.14236 29023711

[B69] Wang B. Wu L. Chen J. Dong L. Chen C. Wen Z. (2021). Metabolism pathways of arachidonic acids: mechanisms and potential therapeutic targets. Signal Transduct. Target. Ther. 6 (1), 94. 10.1038/s41392-020-00443-w 33637672 PMC7910446

[B70] Wang J. Q. Liu Y. R. Xia Q. Chen R. N. Liang J. Xia Q. R. (2019). Emerging roles for NLRC5 in immune diseases. Front. Pharmacol. 10, 1352. 10.3389/fphar.2019.01352 31824312 PMC6880621

[B71] Wang K. C. Fan L. W. Kaizaki A. Pang Y. Cai Z. Tien L. T. (2013). Neonatal lipopolysaccharide exposure induces long-lasting learning impairment, less anxiety-like response and hippocampal injury in adult rats. Neuroscience 234, 146–157. 10.1016/j.neuroscience.2012.12.049 23298854 PMC3594355

[B72] Xie Z. Ma X. Ji W. Zhou G. Lu Y. Xiang Z. (2010). Zbtb20 is essential for the specification of CA1 field identity in the developing hippocampus. Proc. Natl. Acad. Sci. U. S. A. 107 (14), 6510–6515. 10.1073/pnas.0912315107 20308569 PMC2851958

[B73] Young G. B. Bolton C. F. Austin T. W. Archibald Y. M. Gonder J. Wells G. A. (1990). The encephalopathy associated with septic illness. Clin. Invest. Med. 13 (6), 297–304. 2078909

[B74] Zhang Y. Yuan S. Pu J. Yang L. Zhou X. Liu L. (2018). Integrated metabolomics and proteomics analysis of Hippocampus in a rat model of depression. Neuroscience 371, 207–220. 10.1016/j.neuroscience.2017.12.001 29237567

[B75] Zheng Y. Zhou X. Wang C. Zhang J. Chang D. Zhuang S. (2022). Effect of dendrobium mixture in alleviating diabetic cognitive impairment associated with regulating gut microbiota. Biomed. Pharmacother. 149, 112891. 10.1016/j.biopha.2022.112891 35367768

