# Supplementary figures and images for: Integrated transcriptomics and metabolomics analysis of the hippocampus reveals altered neuroinflammation, downregulated metabolism and synapse in sepsis-associated encephalopathy
Source: Front Pharmacol. 2022 Sep 6;13:1004745. doi: 10.3389/fphar.2022.1004745 (PMC9486403; doi:10.3389/fphar.2022.1004745)

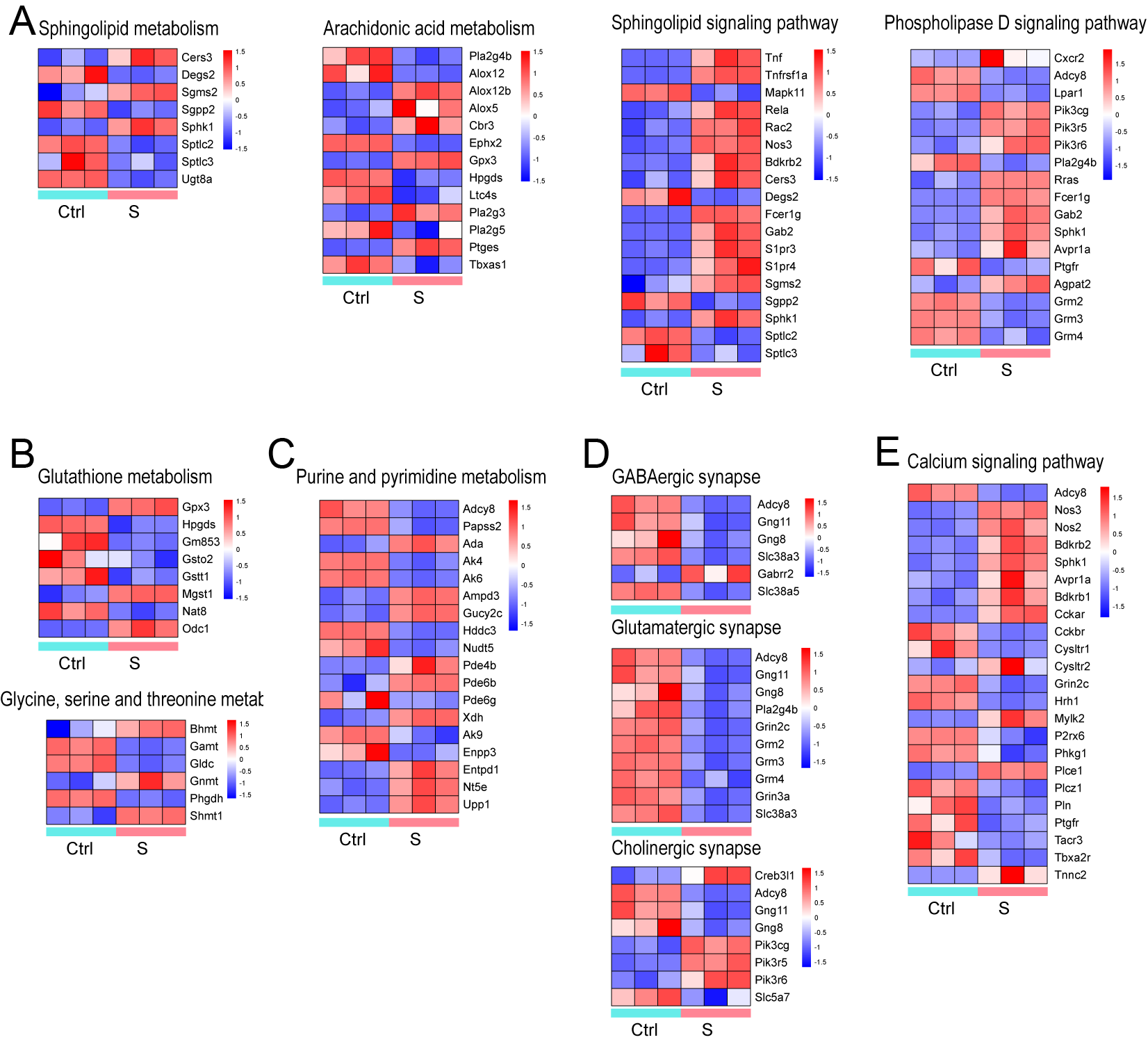

Supplement: Supplementary file 3 [file Image2.TIF]

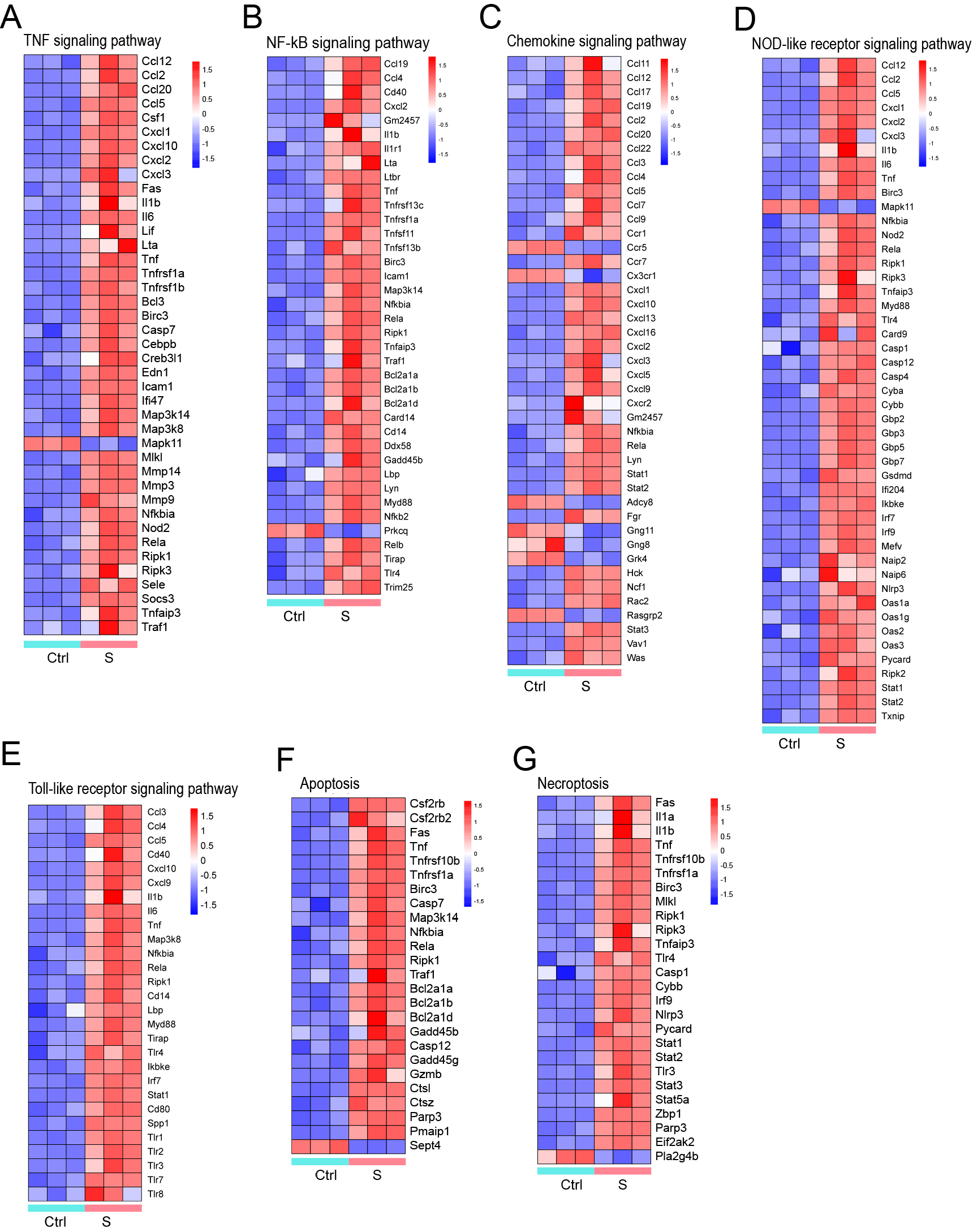

Supplement: Supplementary file 4 [file Image1.TIF]
